# Retrieval-augmented generation improves precision and trust of a GPT-4 model for emergency radiology diagnosis and classification: a proof-of-concept study

**DOI:** 10.1007/s00330-025-11445-z

**Published:** 2025-02-14

**Authors:** Anna Fink, Johanna Nattenmüller, Stephan Rau, Alexander Rau, Hien Tran, Fabian Bamberg, Marco Reisert, Elmar Kotter, Thierno Diallo, Maximilian F. Russe

**Affiliations:** 1 Department of Diagnostic and Interventional Radiology, Medical Center University of Freiburg, Faculty of Medicine, University of Freiburg, Freiburg, Germany; 2 Department of Neuroradiology, Medical Center University of Freiburg, Faculty of Medicine, University of Freiburg, Freiburg, Germany; 3 Department of Stereotactic and Functional Neurosurgery, Medical Center University of Freiburg, Faculty of Medicine, University of Freiburg, Freiburg, Germany

**Keywords:** Artificial intelligence, Deep learning, Emergency medicine, Tomography, Natural language processing

## Abstract

**Objectives:**

This study evaluated the effect of enhancing a GPT-4 model with retrieval-augmented generation on its ability to diagnose and classify traumatic injuries based on radiology reports.

**Materials and methods:**

In this prospective proof-of-concept study, we used retrieval-augmented generation as a zero-shot learning approach to provide expert knowledge from the RadioGraphics top ten reading list for trauma radiology to the GPT-4 model, creating the context-aware TraumaCB. Radiological report findings of 50 traumatic injuries were independently generated by two radiologists. The performance of the TraumaCB compared to the generic GPT-4 was evaluated by three board-certified radiologists, assessing the accuracy and trustworthiness of the chatbot responses in the 100 reports created.

**Results:**

The TraumaCB achieved 100% correct diagnoses, 96% correct classification, and 87% correct grading, outperforming the generic GPT-4 with 93% correct diagnoses, 70% correct classification, and 48% correct grading. TraumaCB sources consistently achieved a median rating of 5.0 for explanation and trust. Challenges encountered mainly involved traumatic injuries lacking widely accepted classification systems.

**Conclusion:**

Augmenting a commercial GPT-4 model with retrieval-augmented generation improves its diagnostic and classification capabilities, positioning it as a valuable tool for efficiently assessing traumatic injuries across various anatomical regions in trauma radiology.

**Key Points:**

***Question*** *Retrieval-augmented generation has the potential to enhance generic chatbots with task-specific knowledge of emergency radiology*.

***Findings*** *The TraumaCB excelled in accuracy, particularly in injury classification and grading, and provided explanations along with the sources used, increasing transparency and facilitating verification*.

***Clinical relevance*** *The TraumaCB provides accurate, fast, and transparent access to trauma radiology classifications, potentially increasing the efficiency of image interpretation in emergency departments and enabling customized reports based on local or individual preferences*.

## Introduction

In an era of rapidly advancing radiological examination techniques, the workload of radiologists continues to increase. This trend is particularly apparent in trauma radiology, as the decreasing time required for a computed tomography (CT) scan has shifted primary evaluation from the operating room to rapid imaging assessment [1]. In particular, the correct classification and grading of traumatic injuries is of paramount importance, as it often determines the choice between conservative and operative treatment [2].

As the field of trauma surgery continues to grow and there are a multitude of radiologic classification systems in trauma radiology, each associated with complex injuries involving different body parts, it is increasingly difficult for practitioners to be familiar with each system and grading. A potential solution lies in the use of large language models (LLMs), such as the widely recognized, recently introduced generative pre-trained transformer (GPT)-4 Turbo by OpenAI [3]. These chatbots excel at efficiently summarizing large amounts of accessible information and offer a promising way to help radiologists meet the growing demands of their profession.

The potential of GPT-4 in trauma radiology has been shown in several research studies, highlighting its ability to support radiologic exam planning [4–6], fracture classification [7], and generating an impression based on findings in free-text reports [8]. While this is promising, the value in diagnosing and classifying conditions across all body parts and the full spectrum of trauma radiology has not yet been investigated.

As the accuracy of GPT-4 Turbo heavily relies on the quality and quantity of the data it was trained on [9], its non-curated database alone could not be sufficient to handle such a complex task. Moreover, the database is currently limited to December 2023 [3]. Higher-order tasks and the categorization of radiological image descriptions have posed a significant challenge in prior research [10]. Even correct chatbot answers can be subject to inaccuracies and hallucinations [10], and the lack of transparency in the sources used raises concerns about accountability [9, 11]. Retrieval-augmented generation (RAG) is an approach that increases LLM performance by introducing task-specific knowledge into each prompt, giving the LLM indirect access to controllable and updatable external knowledge sources. This leverages the chatbot’s ability to generalize its pre-training through semantic similarity processing and potentially leads to more precise, expertly grounded answers [6, 7].

This study aimed to evaluate how enhancing OpenAI’s GPT-4 Turbo with RAG can improve its ability to diagnose and classify traumatic injuries based on trauma radiology reports.

## Methods

Ethical approval was waived for this prospective proof-of-concept study conducted on synthetic data.

### Dataset preparation and indexing

To evaluate the potential of a retrieval-augmented GPT-4 Turbo for diagnosing and classifying traumatic injuries, two experienced radiologists (A.F. and S.R.) independently created written report findings based on 50 of traumatic diagnoses, resulting in a total of 100 reports (datasets 1 and 2). This approach was designed to assess the chatbots’ ability to handle variations in phrasing, word choice, and nuance introduced by different human authors. The reports in both datasets comprised only imaging findings, which were carefully curated to avoid diagnostic interpretations, and omitted the impression section, which typically provides the diagnosis and classification. The reports included either conventional radiography, CT, or magnetic resonance imaging (MRI) as imaging modalities and encompassed a diverse range of randomly selected traumatic injuries from all anatomical regions of the body. This ensured to encompass a wide variety of diagnoses and classification systems featured in the RadioGraphics top ten reading list, reflecting the complexity encountered in real-world clinical practice.

While most reports represented a traumatic injury with a widely accepted radiologic classification system, some defied classification, and some would comply with various classification systems. This was specifically intended to expose possible hallucinations in chatbot responses.

### RAG for trauma-specific knowledge

GPT-4 Turbo by Open-AI [3] served as a chatbot basis. To create a task-specific knowledge base in trauma radiology, we used RAG to incorporate content from the RadioGraphics top ten reading list for trauma radiology [12] into the chatbot’s input. This carefully curated reading list of 70 peer-reviewed articles was specifically designed to educate radiology residents on essential aspects of traumatic injuries, including their radiologic manifestations and the primary classification systems used in routine clinical practice.

To make this extensive knowledge accessible to the chatbot, each sentence along with its five preceding and following sentences was converted into a numerical embedding vector index using the OpenAI text embedding model (text-embedding-ada-002-v2) [13]. This embedding strategy converts sentences into numerical vectors that represent their meaning, thus allowing for similarity mapping. For each prompt question, the radiological report description was similarly transformed into an embedding and matched using the cosine similarity approach as a form of zero-shot learning within the LlamaIndex framework (version 0.10.6) [14].

The twenty best matches were extracted, providing context for each precision prompt and ensuring that the chatbot received targeted expert knowledge specifically aligned with the report. A hyperparameter setting of 0.4 was used for temperature to ensure low creativity. This combination of a vector index and precision prompt, and OpenAI’s GPT-4 Turbo, creates the trauma radiology context-aware ChatBot (TraumaCB).

### Two-step approach and prompting

**Fig. 1:**
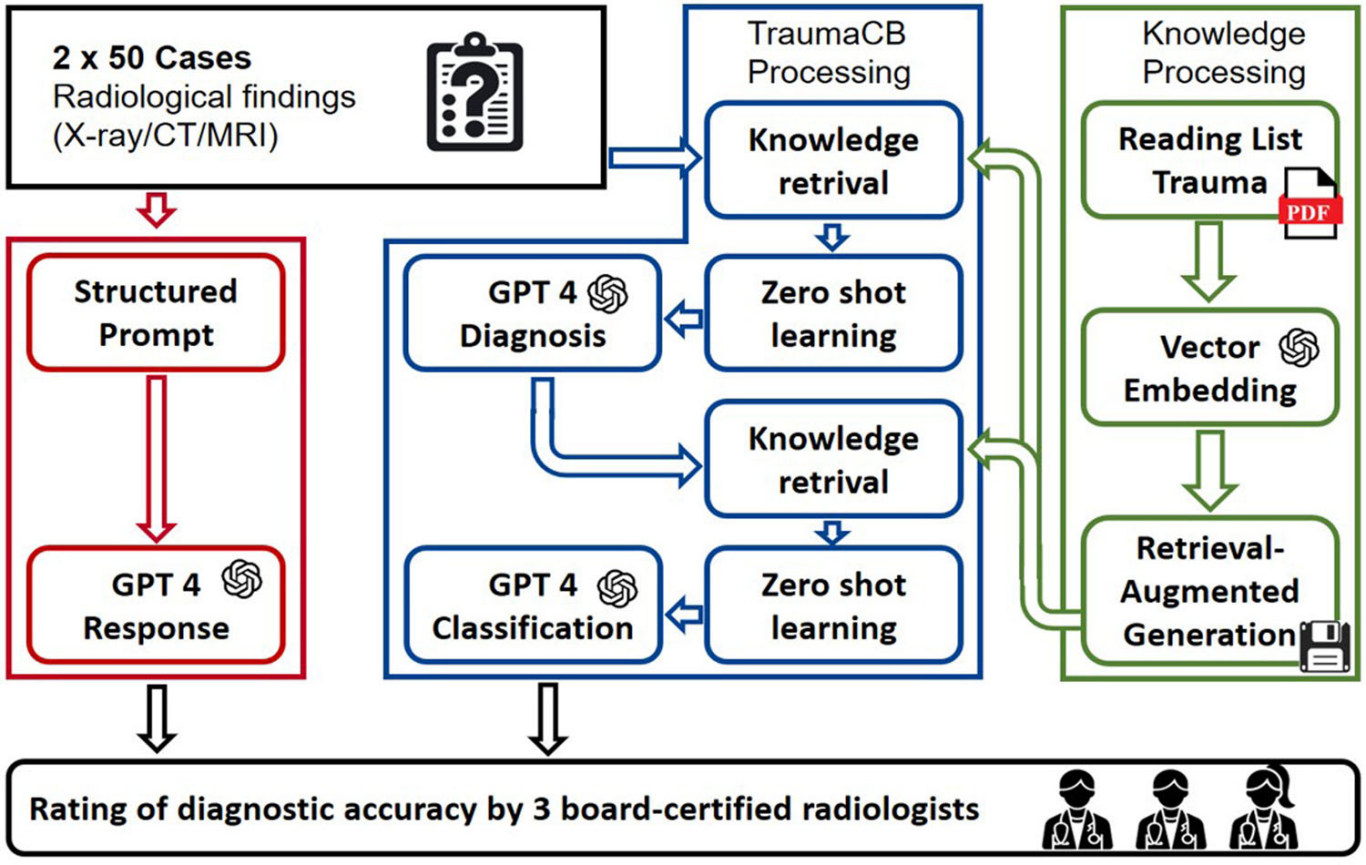
Schematic workflow of the study including report creation, prompting of the generic GPT 4 Turbo, indexing of the top ten reading list for trauma radiology, and the two-step retrieval-augmented approach for the TraumaCB

Within each prompt, we implemented a two-step approach mimicking the clinical workflow, as illustrated in Fig. 1. Initially, the TraumaCB was tasked to provide a primary diagnosis based on the radiological findings and primary context information retrieved from the RadioGraphics top ten reading list. Subsequently, it was presented with the suggested primary diagnosis, radiological findings, and additional expert information from the RadioGraphics top ten reading list specific to the suggested primary diagnosis. The TraumaCB was then instructed to select an appropriate radiological classification system, indicate the correct grading within this system, and provide a concise yet comprehensive explanation.

To ensure a focused response, the chatbot was prompted to select only one classification system and grading, and to explicitly state if no appropriate classification system existed for the given diagnosis. For added transparency, each chatbot response was accompanied by the filenames of the scientific papers that influenced its response. In addition, specific page numbers within these papers were provided, along with hyperlinks to the corresponding pages of the downloaded document PDFs.

To assess the effectiveness of this retrieval-augmented two-step approach, a comparative evaluation was conducted with a comparable precision prompt for the generic GPT-4 Turbo by OpenAI [3]. This prompt did not include any context-specific knowledge and tasked the chatbot solely with providing the primary diagnosis, classification system, grading, and explanation. The detailed two-step input prompt for the TraumaCB can be found in the Supplementary Materials (Suppl. 1).

### Evaluation metrics

For the two datasets of the radiological reports, the chatbot predictions were matched to the ground truth for diagnosis, classification, and grading within the chosen classification system. In addition, three board-certified radiologists not involved in the dataset creation (J.N., T.D., and M.F.R.; all with more than 10 years of clinical experience and subspecialties in musculoskeletal [T.D. and M.F.R.] and emergency radiology [J.N., T.D., and M.F.R.]) evaluated the chatbots’ performance. Evaluation criteria included a subjective rating of trustworthiness and quality of explanation of responses for both the generic chatbot and TraumaCB, as well as correct identification of source files for the TraumaCB. Blinding as to whether the chatbot response originated from the TraumaCB or the generic chatbot was not possible due to the inclusion of sources in the TraumaCB responses. The exact scoring criteria are provided in the Supplementary Materials (Suppl. 2).

Result statistics included median rating scores and interquartile range [IQR] and were calculated using the Pandas software library (version 2.0.3) [15]. A Wilcoxon Signed-Rank Test was used to analyze potential disparities between the ratings using the SciPy software library (Version 1.11.4) [16]. Interrater reliability was assessed using a weighted kappa test across both the explanation and the trust ratings.

### Code and dataset availability

To facilitate open access to the model code, we have created an interactive Jupyter Notebook repository under the MIT license on GitHub based on Python 3.10 mainly using software libraries from Llama Index and OpenAI (https://github.com/maxrusse/TraumaCB).

## Results

### Dataset

The manually curated dataset consists of 100 radiological reports, including 26 conventional radiographs, 68 CT scans (28 contrast-enhanced, 40 unenhanced), and 6 unenhanced MRI scans. Report descriptions featured common traumatic pathologies within musculoskeletal (54%, 54 reports), abdominal (20%, 20 reports), head and neck (14%, 14 reports), thoracic (6%, 6 reports), and pediatric traumatology (6%, 6 reports).

An overview of all traumatic pathologies in the dataset including the corresponding classification system and grading, as well as the imaging modality, is provided in the Supplementary Materials (Suppls. 3), along with exemplary reports from the dataset (Suppls. 4–7).

### Chatbot performance

TraumaCB provided the correct diagnosis in 100% of the 100 radiological reports in the dataset, selected the correct classification system in 96%, and achieved the correct grading within that system in 87% (combined analysis for both datasets). In comparison, the generic GPT-4 Turbo provided the correct diagnosis in 93% of the radiological reports, selected the correct classification system in 70%, and provided the correct grading in 48% (combined analysis for both datasets).

A detailed breakdown of individual performance within each dataset is given in Tables 1–3.

**Table 1:** Comparison of individual chatbot performance within each dataset, evaluating the accuracy of stated diagnoses between the generic GPT-4 Turbo and the TraumaCB

Diagnosis	Generic GPT-4 turbo			TraumaCB		
	Combined	Dataset 1	Dataset 2	Combined	Dataset 1	Dataset 2
Correct (score 5)	93%	88%	98%	100%	100%	100%
Close proximity within the same organ system (score 4)	2%	2%	2%	–	–	–
Wrong (scores 1–3)	5%	10%	–	–	–	–

**Table 2:** Individual chatbot performance within each dataset regarding the stated classification for the generic GPT-4 Turbo and the TraumaCB

Classification	Generic GPT-4 turbo			TraumaCB		
	Combined	Dataset 1	Dataset 2	Combined	Dataset 1	Dataset 2
Correct (score 5)	70%	68%	72%	96%	92%	100%
Close proximity within the same organ system (score 4)	3%	–	6%	2%	4%	–
Wrong (scores 1–3)	27%	32%	22%	2%	4%	–

**Table 3:** Individual chatbot performance within each dataset regarding the grading accuracy within the chosen classification system for the generic GPT-4 Turbo and the TraumaCB

Grading	Generic GPT-4 turbo			TraumaCB		
	Combined	Dataset 1	Dataset 2	Combined	Dataset 1	Dataset 2
Correct (score 5)	48%	46%	50%	87%	86%	88%
Offset of one class (score 4)	26%	28%	24%	12%	12%	12%
Wrong (scores 1–3)	26%	26%	26%	1%	2%	–

Ratings were made on a scale of 1–5, with 1 indicating the lowest and 5 the highest level of trust or explanation. For both datasets combined, the TraumaCB achieved a median score of 5.0 for both explanation and trust, with an IQR of 0.0. The generic GPT-4 Turbo achieved a median score of 4.0 for explanation [IQR 1.0] and 4.0 for trust [IQR 2.0]. The TraumaCB significantly outperformed the generic GPT-4 Turbo on both measures (*p*-value < 0.001). The referenced files accompanying the TraumaCB’s responses closely matched the readers’ expectations in all reports, with a median rating of 5.0 [IQR 0]. Interrater reliability for this subjective assessment demonstrated moderate agreement, with a mean value of 0.59 for both the explanation and trust ratings.

**Fig. 2:**
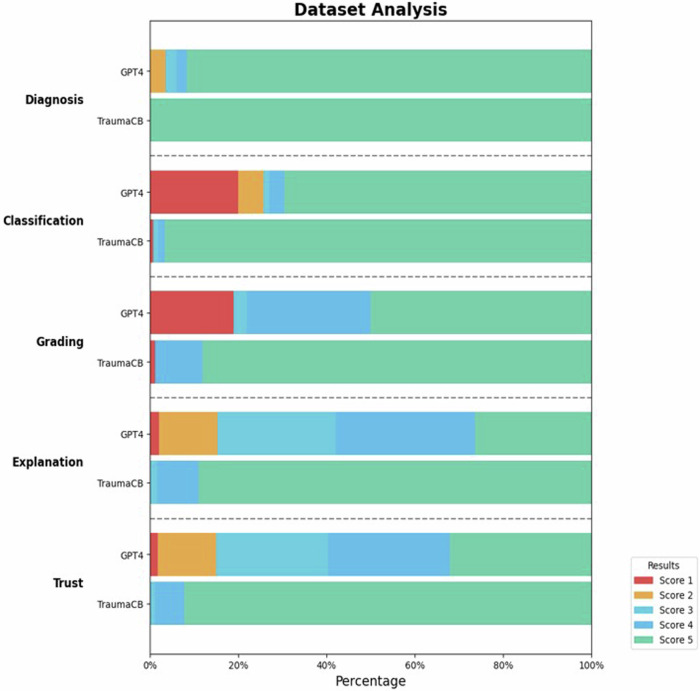
Distribution of the performance of the TraumaCB and generic GPT-4 Turbo with respect to diagnosis, classification, grading, explanation, trust, and sources used (specific for the TraumaCB). Diagnosis scores ranged from 1 (completely wrong) to 5 (correct diagnosis); classification scores ranged from 1 (completely wrong classification system) to 5 (correct classification system); grading scores ranged from 1 (four or more classes off) to 5 (correct grading)

A comprehensive visualization of all results for both the TraumaCB and the generic GPT-4 Turbo is shown in Fig. 2. and a detailed breakdown of the individual scores for each dataset is provided in Table 4.

**Table 4:** Median rating and IQR for the generic GPT-4 turbo and the TraumaCB responses regarding explanation and confidence

	Generic GPT-4 turbo			TraumaCB			*p*-value (combined)
	Complete	Dataset 1	Dataset 2	Combined	Dataset 1	Dataset 2	
Explanation	4.0 [1.0]	4.0 [2.0]	4.0 [1.0]	5.0 [0.0]	5.0 [0.0]	5.0 [0.0]	< 0.01
Trust	4.0 [2.0]	4.0 [2.0]	4.0 [2.0]	5.0 [0.0]	5.0 [0.0]	5.0 [0.0]	< 0.01

Ratings were made on a scale of 1 to 5, with 1 being the lowest and 5 the highest level of trust or explanation

### Targeted case analysis

Reports in which the TraumaCB encountered challenges in providing a concise classification system and grading were rare. This primarily concerned traumatic injuries that can be classified, but for which there are no widely accepted classification systems, including the AAST-classification system for traumatic lung injury, for which the chatbot suggested an alternative classification system (Report 10, Suppl. 5) or stated that no widely accepted radiological classification system exists for this diagnosis (Report 17).

For two reports only, the TraumaCB suggested a classification system not applicable to the traumatic injury. This occurred in the case of an acute subdural hematoma (Report 26) and a Rolando fracture of the first metacarpal bone (Report 31, Suppl. 7), where the chatbot suggested the Kjaer–Petersen classification system, which is typically used for the base of the fifth metacarpal bone. Of note, the chatbot acknowledged this discrepancy in its explanation for both reports. Incorrect grading occurred in three instances, where the TraumaCB provided the correct classification system but was only one grading class away from the ground truth (Reports 15, 19, and 20, Suppl. 6).

In the case of a periprosthetic fracture, the chatbot recognized the lack of clinical information regarding bone stock quality, which is necessary to correctly differentiate between type B2 and B3 in the Vancouver classification (Report 28). The TraumaCB showed no signs of hallucination and correctly identified all other reports where no radiological classification system could be applied (e.g., Reports 2 and 22, Suppl. 3).

## Discussion

As the workload in trauma radiology increases, our chatbot-based approach offers potential assistance through automated diagnosis, classification, and grading of traumatic injuries based on radiological findings. By employing RAG to provide context-specific knowledge to a generic chatbot, we not only achieved correct diagnosis of all traumatic injuries in the dataset, but also significantly improved the accuracy of injury classification and grading compared to the generic GPT-4 Turbo. Trust, transparency, and verifiability were further fostered by providing the scientific sources the chatbot’s response relied on.

Prior research on the capabilities of GPT-4 for clinical decision support in radiology has focused primarily on simplifying radiology reports [17] or enhancing reports in specific contexts [7, 8], leaving the imparting of a broader knowledge base largely unexplored. One notable study exploring a wider spectrum of radiology was conducted by Bhayana et al, who evaluated the performance of GPT-4 in a board-style radiology examination. The authors reported a performance of 69% of questions answered correctly but highlighted the chatbot’s challenges with questions involving higher-order thinking or descriptions of imaging findings [10]. In contrast, our approach demonstrated the ability of a chatbot equipped with task-specific radiological knowledge to accurately identify diagnoses based solely on imaging findings in 100% of the provided reports. This often involved knowledge translation, such as identifying a lentiform hyperdensity located in the left frontoparietal area with a positive swirl sign as a hyperacute epidural hematoma (Report 2).

One of the main advantages of RAG is its ability to access up-to-date and scientifically peer-reviewed sources, as opposed to the non-curated database used to train GPT-4 in its current form. This further avoids binding the chatbot to a static knowledge base, and instead allows the sources used to be adjusted as new or different scientific knowledge emerges.

In this proof-of-concept study, we used two sets of 50 reports, each created independently by a different author, for a total of 100 reports. This approach aimed to introduce variability in language nuances to assess the reproducibility of the models’ results. In addition, a variety of diagnoses and classification systems were included to improve the proofing sample robustness.

We employed a two-step approach to mimic the clinical workflow and to ensure that the chatbot focused its response and retrieved contextual information specific to the most likely diagnosis. This approach further provides the opportunity to gain insight into possible errors in the decision-making process. Future research should evaluate the exact benefit of such multi-step approaches combined with RAG in exploiting specific local dependencies in medical expertise.

Major concerns regarding the integration of LLMs into clinical workflows focus on the lack of explainability and transparency of chatbot responses, largely due to their tendency to confidently state information even when it is incorrect [9–11]. While the TraumaCB outperformed the generic chatbot, it provided slightly inaccurate gradings in three out of the 100 reports. Here, understanding the chatbot’s decision-making might enhance trust and mitigate the risk of overlooking potential misclassifications. We addressed this by including sources, page numbers, and hyperlinks to the scientific papers used alongside each chatbot response, allowing for easy verification of the information provided and enabling the chatbot to be an accessible interactive tool for the radiologist.

Another important concern regarding the integration of LLMs into clinical practice is the risk of hallucination, where chatbots generate false or inaccurate information and confidently present their output as fact [10, 18]. We investigated the performance of our TraumaCB in this regard by intentionally including 16 reports of traumatic injuries for which no radiological classification system exists, e.g., traumatic brain injury (Suppl. 3). Remarkably, the TraumaCB correctly identified almost all of these reports, acknowledging if clinical information was insufficient to accurately grade an injury or no radiological classification system could be applied. In the two reports where the chatbot suggested a classification system inappropriate for the traumatic injury, it however stated this in its explanation (Reports 26 and 31).

A limitation of this study is that we were unable to blind the evaluating radiologists to whether the chatbot response under consideration originated from the TraumaCB or the generic chatbot. This was not feasible due to the inclusion of sources in the TraumaCB responses, potentially leading to confirmation bias in the trust ratings, as radiologists may expect the RAG approach to be more reliable. Additionally, while the RadioGraphics top ten reading list for trauma radiology serves as a reputable, peer-reviewed resource for trauma radiology, it does not encompass the entire field. Our work, however, introduces a framework that can easily be adapted to other knowledge bases. Future enhancements could include the creation of a vector index based on a broader and more diverse collection of resources, such as specialty texts or curated knowledge bases from radiological societies dedicated to this specialty. Given the limited access to OpenAI’s GPT-4, a generic state-of-the-art LLM, and the emergence of new vendors with similar software, such as Gemini Pro [19] or Claude [20], future research should also explore validating the performance of different chatbots in real-world clinical settings. Currently, privacy concerns prevent real patient data from being uploaded to these chatbots, limiting the adoption of such frameworks in routine clinical practice, so addressing this issue is critical. One potential solution could be to use technologies such as Microsoft Cloud for Sovereignty [21], which enables the creation of secure and accessible workspaces while ensuring privacy. However, this technology is not yet widely available. Finally, although this proof-of-concept study employed a dataset of 100 radiology reports, its size remains a limitation. We plan to address this by testing the chatbot on a larger dataset, and expanding its application beyond trauma radiology to further evaluate its performance and versatility.

In conclusion, augmenting GPT-4 Turbo with contextual knowledge enhances its diagnostic classification capabilities in artificially generated radiological reports. The developed chatbot provides quick access to relevant, trustworthy, and transparent information on specific classification systems in trauma radiology. Further advancements should prioritize the real-world applicability of this tool to customize radiology reports, meet the specific needs of departments and referring clinicians, and facilitate rapid radiological assessment.

## Supplementary information

ELECTRONIC SUPPLEMENTARY MATERIAL

## Abbreviations

CT: Computed tomography
GPT: Generative pre-trained transformer
IQR: Interquartile range
LLM: Large language model
MRI: Magnetic resonance imaging
RAG: Retrieval-augmented generation
TraumaCB: Trauma radiology context-aware ChatBot
