# Supplementary material for: Retrieval-augmented generation improves precision and trust of a GPT-4 model for emergency radiology diagnosis and classification: a proof-of-concept study
Source: Eur Radiol. 2025 Feb 14;35(8):5091–8. doi: 10.1007/s00330-025-11445-z (PMC12226682; doi:10.1007/s00330-025-11445-z)
Supplement: Supplementary file 1 — ELECTRONIC SUPPLEMENTARY MATERIAL [file 330_2025_11445_MOESM1_ESM.pdf]

Retrieval-Augmented Generation improves Precision and Trust of a GPT-4 model for Emergency Radiology Diagnosis and Classification: A Proof-of-Concept Study

ELECTRONIC SUPPLEMENTARY MATERIAL

Suppl. 1: Detailed input prompt for the two-step retrieval-augmented approach.

The prompts have been formatted for better readability, with explained variables in [square brackets]. The complete code is available in the publicly accessible Jupyter notebook.

First Step:

We have provided scientific context information below [*Automatically retrieved Top 15 text nodes from the RadioGraphics Top 10 Reading List for Trauma Radiology*]. Given this information, please evaluate the following image finding: [Report *description with imaging findings*].

Please follow the structure below for your response:

- Primary diagnosis (Main pathologic finding as working diagnosis, no explanation)

Second Step:

We have provided scientific context information below [*Automatically retrieved Top 15 text nodes from the RadioGraphics Top 10 Reading List for Trauma Radiology*]. Given this information, please evaluate the following image finding and suggested primary diagnosis: [Report *description with imaging findings and primary diagnosis from the first step*]

Please follow the structure below for your response:

- Primary diagnosis (Main pathologic finding as working diagnosis, no explanation)

- Radiological classification system for this diagnosis (Please state only one classification! If there is no appropriate classification, specify this)

- Grading in the classification system for this diagnosis (Please state only one grade! If there is no classification system, specify this)

- Explanation: Provide a concise yet comprehensive and self-contained explanation summarizing the key points of the main diagnosis, classification system and grading.

Suppl. 2: Rating criteria for the evaluation of chatbot performance in terms of provided explanation, trust, and sources used for the TraumaCB.

Explanation:

- Poor **(1):** Lacks clarity, contains inaccuracies, or irrelevant.
- Fair **(2):** Somewhat clear but lacks detail or has minor inaccuracies.
- Good **(3):** Clear and accurate, covers basic aspects of the case.
- Very Good **(4):** Detailed and accurate, with relevant additional information.
- Excellent **(5):** Comprehensive, highly accurate, enhances understanding deeply.

Trust:

- Very Low **(1):** Significant inaccuracies, unreliable.
- Low **(2):** Some issues affecting reliability and accuracy.
- Moderate **(3):** Generally reliable and accurate with minor issues.
- High **(4):** Largely accurate and coherent, instills high confidence.
- Very High **(5):** Exceptionally accurate, well-supported, very high confidence.

Sources:

- No Match **(1):** The top source's topic is completely unrelated to the specified topic.
- Low Match **(2)**: The top source's topic has a minimal relation to the specified topic.
- Moderate Match **(3):** The top source's topic is somewhat related to the specified topic.

- High Match **(4)**: The top source's topic is very closely related to the specified topic.
- Perfect Match **(5):** The top source's topic aligns perfectly with the specified topic.

Suppl. 3: Compilation of all traumatic pathologies in the dataset including the corresponding classification and grading, if applicable, as well as the imaging modality.

**Report** Nr.	Diagnosis (classification and grading, if applicable)	Modality
1/51	Penetrating small bowel and mesenteric injury (AAST IV)	CT
2/52	Traumatic EDH without herniation	CT
3/53	Proximal femur fracture (Garden I, Pauwels I)	Radiograph
4/54	Blunt liver trauma with laceration (AAST III)	CT
5/55	Laryngotracheal injury (Schaefer-Fuhrman II)	CT
6/56	Complete vertical sacral fracture (Denis zone 2, Isler II)	CT
7/56	Pediatric wrist fracture (Salter Harris III)	Radiograph
8/58	Muscle contusion with hemorrhage	MRI
9/59	Pelvic fracture with venous bleeding (Young-Burgess LC 1)	CT
10/60	Pulmonary laceration and contusion with pneumothorax (AAST II)	CT
11/61	Hemothorax with active bleeding and displaced rib fracture	CT
12/62	Nasal bone fracture (Stranc and Robertson I/Rhee II)	CT

13/63	Blow-out orbital fracture	CT
14/64	Periprosthetic femoral fracture (Vancouver B2)	Radiograph
15/65	Penetrating small bowel transection (AAST IV)	CT
16/66	Bullet trajectory with liver hematoma (AAST V)	CT
17/67	Pulmonary contusion (AAST III)	CT
18/68	Humerus fracture (Neer 2-part-fracture with greater tuberosity)	CT
19/69	Humerus fracture (Neer 3-part-fracture with greater tuberosity)	CT
20/70	Pediatric wrist fracture (Salter Harris IV)	Radiograph
21/71	Proximal femur fracture (Garden IV, Pauwels III)	Radiograph
22/72	Pancreatic contusion (AAST I)	CT
23/73	Pancreatic laceration (AAST V)	CT
24/74	Blunt liver trauma with subcapsular hematoma (AAST I)	CT
25/75	Osteochondral dome fracture of the talus (Sneppen A)	CT
26/76	Traumatic acute SDH without mass effect	CT
27/77	Traumatic temporal and frontal lobe hemorrhagic contusions	CT

28/78	Periprosthetic fracture with implant loosening (Vancouver B3)	CT
29/79	Periprosthetic fracture (Vancouver Agt)	Radiograph
30/80	Bennett fracture of the first metacarpal bone	Radiograph
31/81	Rolando fracture of the first metacarpal bone	Radiograph
32/82	Nasal bone fracture (Robertson/Stranc III, Rhee III)	CT
33/83	Tibial plateau fracture (two columns, Schatzker IV)	CT
34/84	Tibial plateau fracture (one column, Schatzker II)	CT
35/85	Posterior hip dislocation (Levin 4, Thompson-Epstein 4)	Radiograph
36/86	Femoral head fracture (Pipkin 1)	CT
37/87	Femoral head fracture (Pipkin 3)	CT
38/88	SLAP-lesion of the shoulder (Snyder IIa)	MRI
39/89	Pancreatic laceration (AAST II)	CT
40/90	SLAP-lesion of the shoulder (Snyder III)	MRI
41/91	Proximal femur fracture (Garden III, Pauwels II)	Radiograph
42/92	Humerus fracture (Neer 1-Part Humerus fracture)	Radiograph
43/93	Humerus fracture (Neer 4-Part Humerus fracture)	CT

44/94	Blunt liver trauma with subcapsular hematoma (AAST II)	CT
45/95	Tibial plateau fracture (three columns, Schatzker VI)	CT
46/96	Femoral head fracture (Pipkin 2)	CT
47/97	Pediatric fracture (Salter Harris II)	Radiograph
48/98	Sacral fracture (Denis zone 1, Isler I)	CT
49/99	Nondisplaced talar neck fracture (Hawkins-Canale-Type I)	Radiograph
50/100	Gunshot wound with colon transection (AAST IV)	CT

Suppl. 4: Two exemplary reports of correctly diagnosed, classified, and graded traumatic injuries by the TraumaCB.

Report 2: Traumatic epidural hematoma without brain herniation

Unenhanced CT scan of the head shows a large lentiform hyperdensity located in the left frontoparietal area with positive swirl sign. No extension beyond coronal or lambdoid sutures. There is a small regional mass effect primarily affecting the left parietal lobe with compression of the left lateral ventricle. No evidence of brain herniation or brainstem compression.

TraumaCB's response:

- Primary diagnosis: Epidural hematoma (EDH) with swirl sign

- Radiological classification system for this diagnosis: There is no widely accepted specific radiological classification system for EDH based on CT findings.

- Grading in the classification system for this diagnosis: Not applicable as there is no specific grading system for EDH based on CT findings.

- Explanation: The primary diagnosis is an epidural hematoma (EDH), which is a traumatic accumulation of blood between the dura mater and the skull. The swirl sign indicates active bleeding within the hematoma and is characterized by mixed density within the hematoma on a non-contrast CT scan, suggesting a hyperacute stage of bleeding. EDHs are typically biconvex (lentiform) in shape and do not cross suture lines due to the dural attachment at these sutures. The mass effect is evident by the compression of the left lateral ventricle and localized mass effect on the left parietal lobe. There is no evidence of herniation or brainstem compression, which would be indicative of a more severe injury. While there is no specific radiological classification or grading system for EDH based on CT findings, the clinical management and prognosis may be influenced by factors such as hematoma volume, patient's level of consciousness (e.g., Glasgow Coma Scale score), presence of associated injuries, and signs of mass effect or herniation. The next step in management typically involves neurosurgical evaluation for possible intervention, which may include observation, medical management, or surgical evacuation depending on the size, location, and associated neurological symptoms.

- Filenames: Schweitzer et al. - 2019 - Traumatic Brain Injury Imaging Patterns and .pdf Page: (1573, 1575, 1578, 1579, 1580, 1581, 1582, 1584, 1587, 1591, 1594), Kurihara et al. - 2020 - Temporal Bone Trauma Typical CT and MRI Appearanc.pdf Page: (1160)

Report **22: Pancreatic Contusion (AAST I)**

Axial contrast-enhanced CT shows stranding and thickening of the subcutaneous adipose tissue, consistent with the likely impact site of blunt force trauma to the abdomen. Additional adipose tissue stranding is seen adjacent to the uncinate process of the pancreas, which has an area of indistinct hypoenhancement. The main pancreatic duct is of normal width with no evidence of disruption or involvement. There is no active contrast extravasation. No other parenchymal organ injuries are observed.

TraumaCB's response:

- Primary diagnosis: Pancreatic contusion with associated subcutaneous soft tissue injury

- Radiological classification system for this diagnosis: American Association for the Surgery of Trauma Organ Injury Scale (AAST-OIS)

- Grading in the classification system for this diagnosis: Grade I

- Explanation: The primary diagnosis is a pancreatic contusion, which is a type of pancreatic injury characterized by indistinct areas of parenchymal edema and hypoenhancement on contrast-enhanced CT imaging. The presence of subcutaneous soft tissue stranding and thickening suggests associated injury due to blunt force trauma to the abdomen. The main pancreatic duct appears normal in width with no evidence of disruption, and there is no active contrast extravasation, which excludes higher-grade injuries that involve the main pancreatic duct or show signs of active bleeding. For the classification and grading of pancreatic injuries, the AAST-OIS is commonly used. This system stratifies pancreatic injuries based on the

type of injury (hematoma or laceration), the location of the injury (head, body, or tail), and the presence or absence of main pancreatic duct (MPD) injury. According to the provided information, the injury can be classified as Grade I, which is a minor contusion without ductal injury or tissue loss. Grade I injuries are typically managed conservatively and have a favorable prognosis compared to higher-grade injuries.

- Filenames: Ayoob et al. - 2021 - Pancreatic Trauma Imaging Review and Management .pdf Page: (61, 63, 64, 66, 67, 68, 69, 70), Naeem et al. - 2021 - CT of Penetrating Abdominopelvic Trauma.pdf Page: (1074, 1076), Alexander et al. - 2019 - Multidetector CT Findings in the Abdomen and Pelvi.pdf Page: (1195), Sodagari et al. - 2020 - Imaging Evaluation of Abdominopelvic Gunshot Traum.pdf Page: (1775, 1776), Dreizin und Munera - 2012 - Blunt Polytrauma Evaluation with 64-Section Whole.pdf Page: (624), Sheybani et al. - 2014 - Pediatric Nonaccidental Abdominal Trauma What the.pdf Page: (141), Daly et al. - 2008 - Traumatic Retroperitoneal Injuries Review of Mult.pdf Page: (1578)

Suppl. 5: Exemplary report of a correctly diagnosed traumatic injury, in which the TraumaCB proposed an alternative classification system.

Report 10: Pulmonary laceration and contusion with pneumothorax (AAST II)

Axial contrast-enhanced CT of the chest shows a skin defect in the left thoracic wall, possibly the entry site of a bullet trajectory. There is a large disruption of the subpleural left upper lobe tissue forming a gas-filled ovoid cavity with surrounding patchy airspace opacities. There is a sharp anterior demarcation by the visceral pleura and a collection of air in the pleural space at the entry site. No other lung lobes are affected and there is no distal airway leakage or blood vessel disruption.

TraumaCB's response:

- Primary diagnosis: Pulmonary laceration with associated pneumothorax

- Radiological classification system for this diagnosis: Pulmonary laceration types (Lewis et al. - 2021)

- Grading in the classification system for this diagnosis: Type 3, rib penetration tear

- Explanation: The primary diagnosis of pulmonary laceration with associated pneumothorax is based on the CT findings of a skin defect in the left thoracic wall, which is likely the entry site of a bullet trajectory, and a large disruption of the subpleural left upper lobe tissue forming a gas-filled cavity with surrounding patchy airspace opacities. The sharp anterior demarcation by the visceral pleura and the presence of air in the pleural space support the diagnosis of pneumothorax. The radiological classification system for pulmonary lacerations, as described by Lewis et al. (2021), categorizes lacerations into different types based on their characteristics and associated findings. The grading within this system for the described findings would be a Type 3 laceration, which is a rib penetration tear, characterized by a peripheral air-filled laceration subjacent to the thoracic ribs, commonly associated with pneumothorax. The classification aids in understanding the mechanism of injury, potential complications, and guiding appropriate management.

- Filenames: Gunn et al. - 2014 - Current Concepts in Imaging Evaluation of Penetrat.pdf Page: (1827, 1833, 1839), Lewis et al. - 2021 - Imaging Manifestations

of Chest Trauma.pdf Page: (1324, 1325, 1326, 1327, 1328, 1329, 1330, 1331), Lozano et al. - 2013 - Penetrating Wounds to the Torso Evaluation with T.pdf Page: (354)

Suppl. 6: Exemplary report of a correctly diagnosed traumatic injury where the TraumaCB missed the correct grading by one class.

Report 15: Penetrating small bowel transection (AST IV)

Contrast-enhanced CT images of the abdomen and pelvis show gas foci within the subcutaneous fat, indicating both entry and exit wounds caused by a bullet. Following the trajectory of the bullet, a loop of small bowel shows focal wall thickening surrounded by mesenteric stranding and adjacent pneumoperitoneum. There is no evidence of intravenous contrast extravasation or venous pooling.

TraumaCB's response:

- Primary diagnosis: Bowel injury with associated mesenteric injury due to gunshot wound

- Radiological classification system for this diagnosis: American Association for the Surgery of Trauma (AAST) Organ Injury Scale (OIS) for small bowel injuries

- Grading in the classification system for this diagnosis: Grade III

- Explanation: The primary diagnosis is based on the CT findings which show focal wall thickening of a small bowel loop surrounded by mesenteric stranding and adjacent pneumoperitoneum, indicating a bowel injury with associated mesenteric injury resulting from a gunshot wound. The AAST Organ Injury Scale for small bowel injuries is a widely used radiological classification system that categorizes bowel injuries based on the extent of damage observed on imaging and during surgery. In this case, the presence of focal wall thickening with mesenteric stranding and pneumoperitoneum, but without evidence of intravenous contrast extravasation or venous pooling, suggests a Grade III injury. This grade typically includes a bowel wall injury that extends into the muscularis propria or subserosa without perforation, which appears to be consistent with the described CT findings.

- Filenames: Sodagari et al. - 2020 - Imaging Evaluation of Abdominopelvic Gunshot Traum.pdf Page: (1767, 1778, 1779, 1783), Naeem et al. - 2021 - CT of Penetrating Abdominopelvic Trauma.pdf Page: (1071, 1079), Brofman et al. - 2006 - Evaluation of Bowel and Mesenteric Blunt Trauma wi.pdf Page: (1119, 1123, 1124)

Suppl. 7: The only report in which the TraumaCB proposed a classification system that is not applicable to the traumatic injury.

Report 31: Rolando fracture of the first metacarpal bone

Frontal and lateral radiographs of the thumb show multiple fracture lines at the first metacarpal base extending into the articular surface. Several of the comminuted fragments show dislocation greater than 3 mm. There is marked soft tissue swelling around the carpometacarpal joint of the thumb.

TraumaCB's response:

- Primary diagnosis: Comminuted intra-articular fracture of the first metacarpal base with associated dislocation.

- Radiological classification system for this diagnosis: Kjaer-Petersen classification system.

- Grading in the classification system for this diagnosis: Type IIIb.

- Explanation: The primary diagnosis is a comminuted intra-articular fracture of the first metacarpal base with associated dislocation, which is characterized by multiple fracture lines extending into the articular surface and dislocated fragments. The Kjaer-Petersen classification system is used to categorize intra-articular fractures at the base of the fifth metacarpal, but can be conceptually applied to the first metacarpal as well. This system divides fractures into three types based on the involvement of the articular surface: Type I involves less than 50% of the articular surface, Type II involves approximately 50% of the articular surface, and Type III involves more than 50% of the articular surface. Type III is further subdivided into IIIa (without comminution) and IIIb (with articular surface comminution). Given the described comminution and dislocation in the radiographic findings, the appropriate grade within this classification system would be Type IIIb. This classification and grading are important for guiding treatment decisions, as intra-articular fractures with significant comminution and dislocation often require surgical intervention to restore joint congruity and prevent complications such as osteoarthritis and impaired function.

- Filenames: Wieschhoff et al. - 2016 - Traumatic Finger Injuries What the Orthopedic Sur.pdf Page: (1107, 1108, 1110, 1112, 1113, 1114, 1118, 1119, 1120, 1128), Little et al. - 2014 - Pediatric Distal Forearm and Wrist Injury An Imag.pdf Page: (483), Cockenpot et al. - 2016 - Imaging of Sports-related Hand and Wrist Injuries.pdf Page: (686), Sadro et al. - 2015 - Geriatric Trauma A Radiologists Guide to Imaging.pdf Page: (1276)
